# Maternal Effects on Seed and Seedling Phenotypes in Reciprocal F_*1*_ Hybrids of the Common Bean (*Phaseolus vulgaris* L.)

**DOI:** 10.3389/fpls.2017.00042

**Published:** 2017-01-24

**Authors:** Jugpreet Singh, Jose A. Clavijo Michelangeli, Salvador A. Gezan, Hyungwon Lee, C. Eduardo Vallejos

**Affiliations:** ^1^Department of Horticultural Sciences, University of FloridaGainesville, FL, USA; ^2^School of Forest Resources and Conservation, University of FloridaGainesville, FL, USA; ^3^Plant Molecular and Cellular Biology Program, University of FloridaGainesville, FL, USA

**Keywords:** cotyledon reserves, growth models, non-linear growth equations, resource remobilization, seedling growth transgenerational effects, wild and domesticated accessions

## Abstract

Maternal control of seed size in the common bean provides an opportunity to study genotype-independent seed weight effects on early seedling growth and development. We set out to test the hypothesis that the early heterotrophic growth of bean seedlings is determined by both the relative amount of cotyledon storage reserves and the genotype of the seedling, provided the hybrid genotype could be fully expressed in the seedlings. The hypothesis was tested via comparison of seed weight and seedling growth phenotypes of small-seeded (wild, ~0.10 g) and large-seeded (landrace, ~0.55 g) parents and their reciprocal F_1_ hybrids. Akaike's Information Criteria were used to estimate growth parameters and identify the phenotypic model that best represented the data. The analysis presented here indicates that the hybrid embryo genotype is not fully expressed during both seed and seedling growth and development. The analysis presented here shows that seed growth and development are controlled by the sporophyte. The strong similarity in seed size and shape of the reciprocal hybrid seed with seeds of the maternal parents is evidence of this control. The analysis also indicates that since the maternal sporophyte controls seed size and therefore the amount of cotyledon reserves, the maternal sporophyte indirectly controls early seedling growth because the cotyledons are the primary nutrient source during heterotrophic growth. The most interesting and surprising results indicated that the maternal effects extended to the root architecture of the reciprocal hybrid seedlings. This phenomenon could not be explained by seed size, but by alterations in the control of the pattern of gene expression of the seedling, which apparently was set by a maternally controlled mechanism. Although seed weight increase was the main target of bean domestication, it also had positive repercussions on early-growth traits and stand establishment.

## Introduction

Seed size reflects to a great extent the amount of stored nutrient reserves, and as such it has a significant impact on early seedling growth and development (Kitajima, 2003; Zhang et al., 2008; Elwell et al., 2011; Slot et al., 2013). Overall, seedling establishment is controlled by genetic and environmental factors. Among the genetic factors are the genes expressed in the maternal parent that affect seed growth and development in different ways, the genes that control seed size and are expressed in the embryo during seed development, and the genes expressed in the seedling itself. Environmental factors refer to those that affect the maternal plant during seed development, seed germination, and seedling growth during the heterotrophic and mixed phases of development. The environmental factors make it possible for an inbred genotype to produce a range of seed size phenotypes as first demonstrated by Johannsen (1911), and this type of non-genic variation may also have a significant effect on early heterotrophic seedling growth (Elwell et al., 2011). These observations indicate that identifying the genic and non-genic factors that control seed size is an essential requirement for identifying and understanding the factors that affect early seedling growth and development.

Seed development begins after sperm cells from the male gametophyte fuse with the egg cell and central cells to form the embryo and the endosperm, respectively. The embryo (n ♀ + n♂) and endosperm (2n♀ + n♂) constitute the filial components of the developing seed, while the maternally derived ovule integuments form the seed coat (2n♀). Seed growth and development depends on the temporal patterns of gene expression of these three structures, each with a different genomic constitution. The maternal seed coat plays a defining role in embryo growth and development by controlling the flow of carbon, nitrogen, minerals (Weber et al., 2005; Zhang et al., 2007), and the extent of cell division (Davies, 1975; Weber et al., 1996; Lemontey et al., 2000). In legumes, the endosperm occupies the largest part of the post-fertilization embryo sac providing nutrients and energy to the developing embryo, and it is the main tissue that exerts maternal effects during early seed development. The endosperm is ephemeral in this group of plants, and it is completely consumed before the start of the embryo growth phase (Hedley and Ambrose, 1980). For these reasons, it has been suggested that some maternal effects occur during the early phases of seed development and could be expressed through the endosperm. Maternal effects in general have been defined by Wolf and Wade (2009) as “the *causal* influence of the maternal genotype or phenotype on the offspring phenotype.”

The maternal parent has been reported to add a certain level of variation to seed size that is independent from that exerted by either the cytoplasmic genomes or the genome of the embryo (Roach and Wulff, 1987). Several examples have been reported recently on transgenerational maternal effects on the phenotype of the progeny, some appear to be induced by abiotic and biotic stimuli, while others are not. For instance, it has been reported in *Campanulastrum americanum* that the maternal light environment has a significant effect on the fitness of the next generation when grown under the same light environment (Galloway and Etterson, 2007). Biotic stimulus can also lead to transgenerational effects as shown by the increase in resistance to *Fusarium* in progeny of maternal plants of *Pinus pinaster* exposed to the pathogen (Vivas et al., 2013). Maternal effects have also been observed in reciprocal hybrids of *Potamogeton* species, which display both, anatomical and physiological differences resembling those between the maternal progenitors (Iida et al., 2013). Similarly, seed size differences in reciprocal hybrids of *Pisum* have been reported in which the hybrid seeds resemble the maternal seed size (Davies, 1975), a phenomenon observed in many legumes. In summary, variation in seed size can be explained by complex genetic and maternal components and their interactions. The maternal component refers to (a) the mechanism governing the parent-of-origin effect by which the maternal genome attempts to maximize its fitness by ensuring equal distribution of resources to all the developing embryos it is bearing, and is explained, at least in part, by the parental conflict theory (Moore and Haig, 1991); and (b) the genetic mechanism that operates in the developing embryo. At the same time the environment could exert a direct and immediate effect through nutrient availability, temperature, and through the effect on the maternal mechanism that controls the parent-of-origin effect.

The goals of this study were to evaluate the effect of seed size on early heterotrophic seedling growth of a single genotype, with particular emphasis on root growth and development, and to assess the extent of parent-of-origin effect on seedling growth and development. Comparing small-seeded with large-seeded varieties carries the risk of confounding the effects of genes that control seed and seedling size with those of unrelated genes that control growth and developmental rates. To study the effect of seed size on seedling growth of the same genotype one could alter seed size by manipulating growth conditions of the maternal plant during seed development, but this treatment may alter embryo development and the subsequent performance of the seedling. The ideal material for our intended study would be two sets of seeds of the same nuclear genotype, but with contrasting seed size phenotypes produced under the same optimal environmental conditions. This ideal material can be obtained by simultaneously generating reciprocal F_1_ crosses between large and small seeded genotypes of the common bean (*Phaseolus vulgaris* L.)—landrace G19833 (~0.55 g/seed) and wild accession G23419 (~0.10 g/seed). As in the case reported for *Pisum* (Davies, 1975), the hybrid seed will have exactly the same nuclear hybrid genotype, but will show contrasting seed size phenotypes resembling those of their maternal progenitors. We hypothesized that comparative analyses of parents with contrasting seed weights and their reciprocal F_1_ hybrids could yield this information, provided the hybrid genotype can be fully expressed in the seedlings. With this material at hand, we hypothesized that the early heterotrophic growth of seedlings is determined by both the relative amount of cotyledon storage reserves and the genotype of the seedling. Accordingly, seedling growth traits that depend on the amount of nutrients stored in the cotyledons will be similar between seed size phenotypes, and growth characteristics controlled by the genotype of the seedling will display differences between parents and hybrids. The current study evaluates this hypothesis with rigorous testing carried out with a comprehensive time-series analysis of root growth and architectural traits along with leaf traits using non-linear equations. Differences in equation parameters between genotypes revealed the extent of maternal effects on root and shoot phenotypes during early growth.

## Materials and methods

### Plant materials

A landrace (G19833) of the common bean, *Phaseolus vulgaris* L., and a wild accession (G23419), both from the Andean gene pool, were selected as the parental genotypes. These accessions display a threefold difference in seed size, and therefore, represent the ideal material for studying the effect of seed size on early root growth and development. The average single seed weight of these genotypes is 0.56 and 0.18 g for G19833 and G23419, respectively (Table 1). To avoid any potential environmentally related transgenerational maternal effects, parental seeds were obtained by growing plants under the same conditions for two generations. Reciprocal F_1_ progenies were obtained by artificially pollinating pistils of emasculated maternal parents with pollen from the male donor. Seeds from all four lines (two parental and two reciprocal F_1_'s) were used to characterize various root and shoot growth features. Two different experiments were performed. In the first experiment, early root and shoot growth of parental and reciprocal lines was monitored without any destructive sampling as described below. In the second experiment, destructive sampling was used to observe the decay in cotyledon dry weight and increase in seedling dry weight over time.

**Table 1:** **Mean seed size and shape parameters values of the parental genotypes G19833 and G23419 and their reciprocal F_1_ hybrids**.

**Genotype**	**Weight (g)**	**Length (mm)**	**Width (mm)**	**Height (mm)**	**Area (cm^2^)**	**Perimeter (cm)**	**Circularity**	**Aspect ratio**
G19833	0.55a	7.71a	5.88a	15.6a	1.20a	4.90a	0.64a	1.89a
	(0.01)	(0.08)	(0.06)	(0.15)	(0.02)	(0.09)	(0.02)	(0.02)
G19833 × G23419	0.52a	7.63a	5.87a	15.2a	1.19a	4.86a	0.61a	1.90a
	(0.01)	(0.08)	(0.06)	(0.15)	(0.02)	(0.09)	(0.02)	(0.02)
G23419 × G19833	0.101b	5.37b	2.94b	7.8b	0.39b	2.54b	0.77b	1.48b
	(0.01)	(0.08)	(0.06)	(0.15)	(0.02)	(0.09)	(0.02)	(0.02)
G23419	0.10b	5.28b	2.98b	7.7b	0.40b	2.56b	0.76b	1.48b
	(0.01)	(0.08)	(0.06)	(0.15)	(0.02)	(0.09)	(0.02)	(0.02)
*p*-value	<0.001	<0.001	<0.001	<0.001	<0.001	<0.001	<0.001	<0.001

*Standard errors of the means are in parenthesis. Different letters indicate significant differences using Tukey's HSD test with α = 0.05*.

### Growth conditions

Seeds were weighed individually, and imbibed in water for 6 h, a period after which the seed coat was removed and seeds were left to imbibe continuously overnight in the dark at 25°C. After imbibition, seeds were germinated in germination paper rolls. This approach allowed us to germinate several seeds and choose the most uniform subset among them for growth analysis. Imbibed seeds were individually rolled in 15 × 15 cm germination paper and placed ~1.25 cm from the upper edge and oriented with the hylum closest to the upper edge and the micropyle toward the longest stretch of paper. This arrangement ensured the radicle would grow downward from the start. The paper roll was in turn rolled into a Mylar sheet, and the entire roll was secured with a plastic band. The rolls were placed vertically on the pegs of polypropylene test tube/drying racks with the seed-side up. The bottom part of the roll was submerged in Hoagland solution. After germination, the seedlings were allowed to grow until the radicle reached a length of 9–10 cm. At this point, the seedlings were transferred to root plates where the emerging radicle was placed between a glass plate (43 × 33 × 0.2 cm) and a sheet of germination paper of equal size (Anchor Paper Inc.). A sheet of Mylar was placed over the filter paper to prevent evaporation. The germination paper, Mylar and a sheet of foam board were secured onto the glass plate by means of paper binders. The plates were positioned in plate racks placed at the bottom of black HDPE boxes (L 58.4 cm × W 38.1 cm × H 45.7 cm; National Tank Outlet, Memphis, TN). Glass plates were placed vertically on custom-built racks. The bottom 2.5 cm of the glass plates was submerged in nutrient solution, which reached the roots by capillary action through the germination paper. The emerging hypocotyls were exposed to light by directing them out through holes in the tank lid. This setup facilitated growing roots in the dark, while the shoots were exposed to light. The tanks were placed in growth chambers under a photon flux density of 400 μmol m^−2^ s^−1^ and a 25°/18°C thermoperiod that was synchronized with a 12 h photoperiod. Identical growth chamber conditions were used for the destructive sampling experiment.

### Growth measurements and data collection

#### Seed characteristics

Seed measurements were taken on 30-seed samples from each of the parents and the reciprocal F_1_ hybrids. Individual seed weight was obtained with an analytic balance to a resolution of 0.1 mg. After imbibition, seed coats were removed, dried and weighed. For all future calculations, seed weights excluded the dry weight of the seed coat. Seed length (SLEN, mm), width (SWID, mm), and height (SH, mm) were measured with a Vernier caliper at a resolution of 0.1 mm. The width represents the maximum distance between the front of the seed with the hilum to the opposite side, while the thickness represents the distance between the lowest and the highest points of the seed when it lays down on a horizontal surface (also known as height). Seed area (SA, mm^2^) and seed perimeter (SPER, mm) were measured by taking images of seeds using a known scale. The images were evaluated using ImageJ software (Rasband, 1997-2011). The ImageJ output provided seed area (SA, cm^2^), seed perimeter (SPER, cm), circularity [shape parameter calculated as 4π [Area/perimeter ^2^], and is defined using a value from 0 (elongated) to 1 (complete circle)], and the aspect ratio (AR) defined as the major against minor axis ratio when an ellipse is fitted to the seed shape.

#### Root traits

Root images were collected non-destructively using an Epson scanner. Roots grown on glass plates were scanned every other day after transplanting up to 12 days post germination. The software package WinRhizo Pro9a (Regent Inc. Canada) was used to obtain measurements of total root length (TRL, cm) and number of branches (FORK, count). Primary root length (PRL, cm) and average basal root length (AvBRL, cm) were calculated manually using the ImageJ software. A centimeter scale was used as a reference to count the number of pixels per centimeter and to calculate the length of primary and basal roots using a free hand drawing tool. Each studied trait represents a distinct class of root growth. For instance, TRL represents root size, FORK represents the branching pattern, and PRL and AvBRL define the framework of a root system.

#### Shoot traits

Hypocotyl and epicotyl diameters were measured at the end of the experiment with a Vernier caliper with a 0.1 mm resolution. Total leaf area (LAREA, cm^2^) and leaf perimeter (LPER, cm) were measured over time using a portable leaf area meter (Li-COR, Nebraska).

#### Dry weight measurements

Cotyledon and seedling tissues were collected at 2-day intervals for dry weight measurements. These tissues were oven dried at 60°C for 72 h, and weighed after the dried tissues equilibrated to room temperature. The measurements were used to evaluate the dynamics of seedling dry weight (SDLDW, g) and cotyledon dry weight (CDW, g).

### DNA extraction and molecular marker analysis

The genotype of the reciprocal F_1_ hybrids was tested with a polymorphic DNA marker. Following phenotypic analysis, a small disc sample from leaf tissue was obtained for DNA extraction from the parents and the putative F_1_ individuals. A leaf disc was ground in a 1.5 ml microcentrifuge tube into a fine powder in liquid nitrogen with a suitable pestle. The powder was resuspended in 800 μl of sample resuspension buffer [100 mM Tris.HCl (pH 8), 50 mM NaCl, 0.5%, Triton X-100, and 1% β-mercaptoethanol]. After vortexing, the resuspension was centrifuged at 1200 rpm for 5 min. After discarding the supernatant, the pellet was resuspended in 250 μl of nuclear resuspension buffer (100 mM Tris.HCl (pH 8), 50 mM NaCl, 0.5%) and 2 μl of freshly boiled RNAse (10 mg/μl) was added and the sample was incubated for 15 min at room temperature. The nuclear fraction was lysed by the addition of 250 μl of warm 2X lysis buffer, and the tubes were incubated for another 45 min at 65°C mixing the lysate by tube inversion every 5 min. The lysate was extracted with 250 μl of chloroform and the tube was chilled first, and then centrifuged at 13,500 rpm for 10 min. The supernatant was transferred to a new tube, and DNA was precipitated with equal volume of isopropanol. The precipitated DNA was pelleted at 13,500 rpm for 5 min, washed in 100% ethanol, dried and dissolved in 0.1X TE buffer.

PCR amplification was carried out with primers designed from a bean sequence (Phvul.005G138300) orthologous to the Arabidopsis *Apetala2* gene. Primer3 (http://www.bioinformatics.nl/cgi-bin/primer3plus/primer3plus.cgi/) was used to design the forward (TTCGATGCTTCTTTGCTATTTTT), and reverse (AAGATCGTGACTGCCACCTT) primers. The PCR mixture contained 2 μl of 1 μM forward and reverse primers; 2 μl of dNTPs (100 μM each of dATP, dGTP, dCTP, and dTTP); 2 μl of 1.5 mM MgCl_2_; 2 μl of 20 ng/μl genomic DNA; and 2 μl Taq DNA polymerase (0.25 U μl^−1^) in a final volume of 20 μl. Thermocycler conditions were 95°C for 2 min, followed by 35 cycles at 95°C for 20 s, 50°C for 30 s, and 72°C for 3 min. The amplification products of parents and putative reciprocal F_1_ hybrids were digested with Taq1 restriction enzyme at 65°C for 1 h and analyzed by electrophoresis in 2% agarose gels.

### Growth model selection and parameter estimation

Data were analyzed using non-linear regression of growth functions with biologically relevant parameters that are associated with different aspects of the growth trajectories. Hence, specific hypothesis can be tested about the genetic control of root and shoot growth processes through rigorous statistical comparisons of the parameters from different genotypes.

An expolinear growth function (Goudriaan and Monteith, 1990) was used to model total root length (TRL, cm) and fork counts (FORK), and it is expressed as follows:

(1) $$\begin{array}{r} Y_{t}=\frac{Cm}{Rm}\;\cdot \log\left[1+e^{Rm\cdot \left(t-tb\right)}\right] \end{array}$$

*Y*_*t*_ represents the response variable at time *t* (days after imbibition); “*Cm*”represents the maximum absolute growth rate, or the amount of response (length, number) per unit time during the linear growth phase; “*Rm*” represents the maximum relative growth rate, or the response change per unit response per day; and “*tb”* (days) is the x-axis intercept for the linear phase of growth.

A modification of the Gompertz growth function (Thornley and France, 2007) was selected for modeling the following growth processes: Primary root length (PRL, cm), average basal root length (AvBRL, cm), leaf area (LAREA, cm^2^) and seedling dry weight (SDLDW, g). The Gompertz growth equation is as follows:

(2) $$\begin{array}{r} Y_{t}=Wf\cdot e^{-e^{-k\left(t-TT\right)}} \end{array}$$

*Y*_*t*_ represents the response variable at time *t* (days after imbibition); “*Wf*” represents the maximum (asymptotic) values for the response variables; “*k*” represents the maximum relative growth rate, or the amount of growth in mass, length, or surface area per unit of time in days, based on each unit of mass, length, or surface (g g^−1^ d^−1^, cm cm^−1^ d^−1^, cm^2^ cm^−2^ d^−1^, or d^−1^); and “*TT”* is the time required to reach the maximum relative growth rate, before the rate begins to decline.

The decline of cotyledon dry weight (CDW, g) was modeled with a decay equation previously used to model competitiveness of ligand-receptor binding (Motulsky and Christopoulos, 2004). The fitted decay equation is as follows:

(3) $$\begin{array}{r} Y_{t}=Min+\frac{Max-Min}{1+e^{\left(t-TT\right)}} \end{array}$$

*Y*_*t*_ is the response variable at time *t* (days after imbibition). “*k”* represents the relative rate of decay; “*Max”* and “*Min*” represent the initial and end point parameter values, respectively; and “*TT*” represents the time at which *Y*_*t*_ reaches the mid-point between “*Max*” and “*Min*”.

### Statistical analyses

Comparisons of growth and developmental parameters were carried out to test our phenotypic control hypotheses. End-point traits (seed weight and dimensions, and hypocotyl and epicotyl diameters) were analyzed using fixed-effects models with genotype as main effects via the *gls* function within the *nlme* package (Pinheiro and Bates, 2000) of the R statistical software (http://www.R-project.org/). Means were compared using Tukey's Honestly Significant Difference at α = 0.05, via the *lsmeans* package (Lenth, 2015).

The hypothesis about the parent-of-origin effect on early seedling growth was statistically tested on five different genetic models: The GEN4 model assumes parameter values were specific to each genotype, distinguishing the reciprocal F_1_s from each other (four levels); the GEN3 model assumed parameter values were specific to each of the three nuclear genotypes (three levels); the MAPHE model assumed parameter values were specific to maternal seed-size (two levels, one for each of large and small seed classes); the MAHY model assumes parameter values are indistinguishable between the parents, but these are different from the reciprocal F_1_ hybrids, which are different from each other (three levels); and the UNI model assumes there were no differences among genotypes (one level). A complete list of each parameter combination for tested models is presented in Table 2 for root and cotyledon/leaf/seedling traits, respectively.

**Table 2:** **Results of the Akaike Information Criteria tests of the five models tested**.

**Parameter grouping**	**df**	**logL**	**AICc**	**ΔAICc**	**wAICc**	**ER**
**TOTAL ROOT LENGTH (TRL**)						
Seed size (MAPHE)	9	−469.8	959.4	0	0.71	–
Genotype (GEN4)	15	−463.0	961.2	1.8	0.29	2.5
Parental (MAHY)	12	−485.5	998.2	38.8	2.67E–09	2.68E+08
Hybrid (GEN3)	12	−487.3	1002	42.6	4.06E–10	1.76E+09
All (UNI)	6	−500.0	1012.9	53.5	1.74E–12	4.09E+11
**NUMBER OF BRANCHES (FORK)**						
Seed size (MAPHE)	9	−576.4	1172.6	0	0.94	–
Genotype (GEN4)	15	−571.5	1178.2	5.6	0.06	16.8
Parental (GEN3)	12	−587.9	1203.0	30.5	2.30E–07	4.11E+06
Hybrid (MAHY)	12	−590.3	1207.8	35.3	2.07E–08	4.57E+07
All (UNI)	6	−601.0	1214.9	42.3	6.03E–10	1.57E+09
**PRIMARY ROOT LENGTH (PRL**)						
Seed size (MAPHE)	9	−88.8	197.4	0	0.97	–
Genotype (GEN4)	15	−84.5	204.3	6.9	0.03	31.1
Hybrid (MAHY)	12	−102.9	233.0	35.6	1.82E–08	5.32E+07
Parental (GEN3)	12	−109.4	246.1	48.7	2.58E–11	3.76E+10
All (UNI)	6	−122.8	258.3	60.9	5.70E–14	1.70E+13
**AVERAGE BASAL ROOT LENGTH (AvBRL)**						
Seed size (MAPHE)	9	−32.7	85.2	0	1	–
Genotype (GEN4)	15	−30.9	97.0	11.8	2.74E–03	363.5
Parental (GEN3)	12	−45.4	118.2	32.9	6.99E–08	1.43E+07
Hybrid (MAHY)	12	−57.8	143.0	57.7	2.92E–13	3.41E+12
All (UNI)	6	−66.2	145.2	59.9	9.70E–14	1.03E+13
**LEAF AREA (LAREA)**						
Seed size (MAPHE)	9	−137.8	297.2	0	1	–
Genotype (GEN4)	15	−134.8	310.6	13.3	1.28E–03	780.3
Hybrid (MAHY)	12	−147.0	324.6	27.4	1.12E–06	8.95E+05
All (UNI)	6	−155.7	325.1	27.8	9.05E–07	1.10E+06
Parental (GPA)	12	−148.3	327.3	30.1	2.97E–07	3.36E+06
**DECAY IN COTYLEDON DRY WEIGHT (CDW)**						
Seed size (MAPHE)	13	116.2	−195.7	0	9.10E–01	13
All (UNI)	10	108.5	−191.0	4.6	9.02E–02	10
Hybrid (MAHY)	16	113.5	−177.5	18.1	1.06E–04	16
Parental (GPA)	16	113.0	−176.5	19.1	6.29E–05	16
Genotype (GEN4)	19	114.1	−163.0	32.6	7.54E–08	19
**SEEDLING DRY WEIGHT ACCUMULATION (SDLDW)**						
Seed size (MAPHE)	9	135.0	−247.3	0	1	–
Genotype (GEN4)	15	138.3	−231.7	15.6	4.10E–04	2437
Parental (GEN3)	12	114.4	−195.8	51.4	6.86E–12	1.46E+11
All (UNI)	6	104.4	−194.8	52.4	4.09E–12	2.44E+11
Hybrid (MAHY)	12	113.2	−193.6	53.7	2.21E–12	4.53E+11

*Selection criteria for genetic models that best explain the phenotypes of reciprocal F1 hybrids. Notations: df, model degrees of freedom; logL, model log likelihood; AICc, sample-size corrected Akaike's information criterion; ΔAICc, difference in AICc values between the best model (lowest AICc) and every other model; wAICc, Akaike weights; ER, evidence ratio, the ratio between the best model wAICc and those of every other model. GEN4 Model, Each parent and reciprocal hybrid possess a significantly different parameter values; GEN3 Model, three parameter classes, the two parents and the hybrid; MAPHE model, parameters are specific to each seed size class. MAHY model, no difference between values of parental lines, but these that are different from the reciprocal hybrids, which are different from each other; UNI model, no significant parameters differences among the genotypes*.

The non-linear growth models (expolinear, Gompertz, and decay) were then fitted to the data using generalized non-linear least squares (*gnls*) as implemented in the *nlme* package (Pinheiro and Bates, 2000) of the R statistical software (http://www.R-project.org/). Model comparisons were done using Akaike's Information Criteria corrected for sample size (AICc) (Burnham and Anderson, 2002), where for each trait, models with the lowest AICc value were selected as best. The AICc estimates the relative Kullback-Leibler distance between the (unknown) true data-generating mechanism and each of the fitted models. Akaike weights (w_AICc_) were calculated for each model, while ΔAICc (difference between the best and every other model) and evidence ratios (ER) were calculated between the best model and all others (Burnham and Anderson, 2004). Akaike weights provide a continuous measure of support for each individual model relative to all others tested on a scale of 0 (no support) to 1 (full support) (Burnham and Anderson, 2004). Δ-AICc values were used to select the most plausible models, with those having Δ-AICc < 2 have strong support, those with Δ-AICc between 4 and 7 substantial support, and any model with Δ-AICc > 10 discarded from consideration. An autoregressive first-order (AR1) covariance structure was employed to capture the correlated nature of time-series observations on the same plants. In addition, the most parsimonious variance structure was determined by comparing different variance functions on the GEN4 model for each trait, using w_AICc_ as selection criteria. For all traits the best covariance structure selected from nlme was *varExp*, an exponential function of the harvest (days) covariate estimated across genotypes, except for cotyledon dry weight decay, which was best modeled by estimating an individual variance term (*varIdent*) for each harvest, across genotypes Additionally, asymptotic 95% confidence intervals were obtained to gauge the variability of parameter estimates.

## Results

### Contrasting seed size phenotype of a single genotype

**Figure 1:**
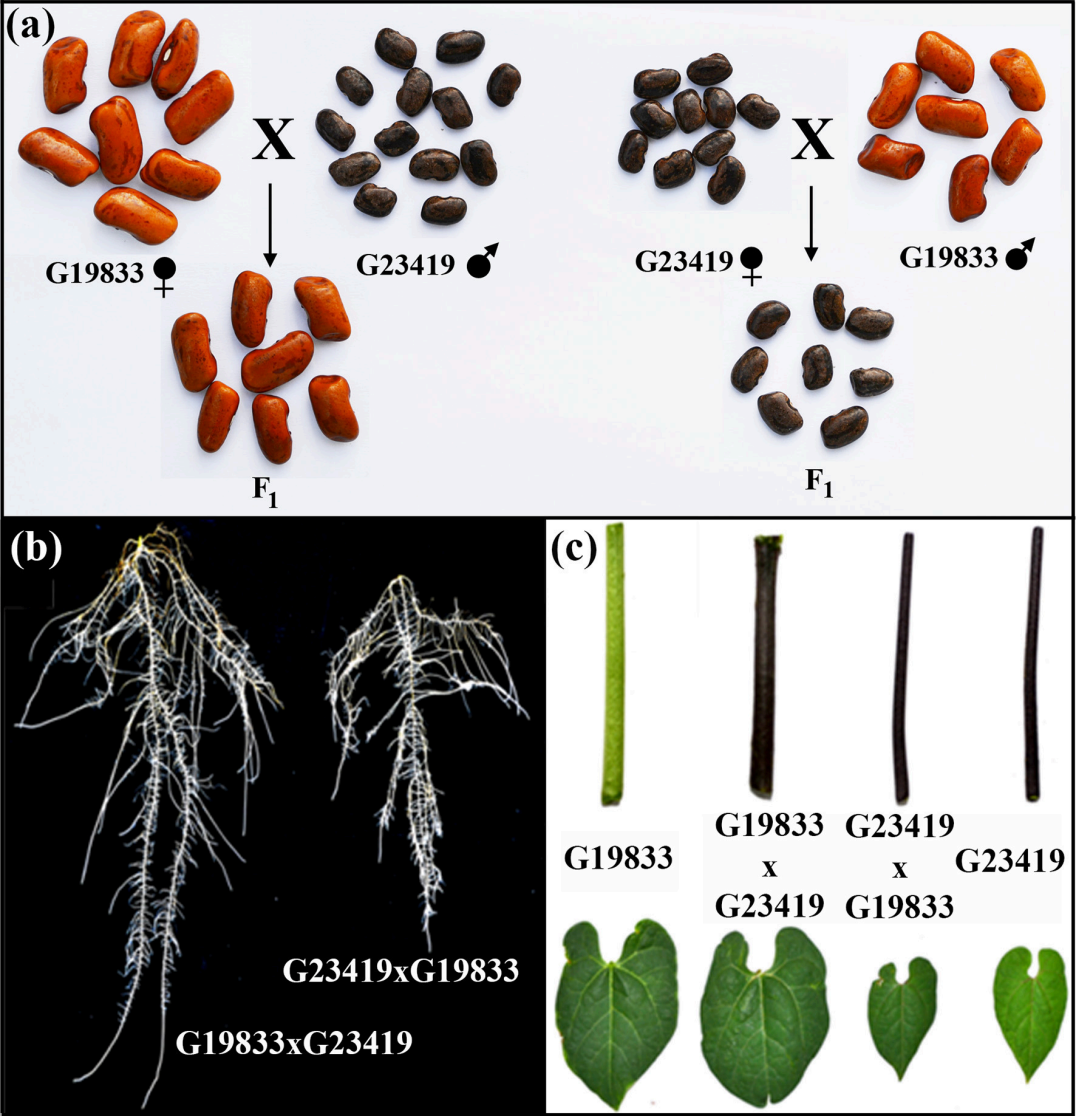
**Phenotypes of the parental lines (landrace G19833, and wild G23419) and their reciprocal F_1_ hybrids: (A)** seeds, **(B)** roots, and **(C)** shoot organs.

The experiments were carried out with seeds of the three different nuclear genotypes, the two parents and the reciprocal F_1_ hybrids. The phenotypes of these seeds were characterized by one- and two-dimensional descriptive parameters of seed size and shape as presented in Table 1. Statistical comparisons by Tukey's honest significant difference (HSD) of the means clearly indicated that the parents possess contrasting seed size and shape phenotypes. Seeds of the landrace G19833 are five times heavier than those of the wild accession G23419, and they are between 1.5 to 2 times larger in one-dimensional measurements than the wild counterpart. Circularity and aspect ratio measurements also indicated that the parental seeds have contrasting shapes as well. Furthermore, these comparisons also indicated that the reciprocal crosses produced F_1_ hybrid seeds that were indistinguishable from seeds produced by selfing of maternal plants (Figure 1). In summary, the three genotypes only displayed two phenotypes; the large seeded maternal parent produced large seeds and the small seeded maternal parent produced small seeds, regardless of the genetic makeup of the pollen donor. Thus, although the reciprocal F_1_ hybrids possess identical nuclear genotypes, they displayed seed phenotypes that were as contrasting as those of the parents. The genotype of the reciprocal hybrids was confirmed via DNA marker analysis (Figure S1). In addition, F_1_ seed harvested from G19833 inherited the dominant hypocotyl pigmentation trait carried by the wild accession G23419 (Figure 1). The reciprocal F_1_ hybrids appeared to be an ideal material to differentiate seed size effects from genetic effects during early seedling growth.

### Comparative analysis of early seedling growth and development between reciprocal hybrids and their maternal parents

We hypothesized that growth parameters associated with organ size during early seedlings growth would be strongly influenced by seed size. For instance, final seedling shoot and root size-specific parameters, defined by the asymptote “*Wf*” [Equation (2)], provide a measure of total root length or leaf area, and are expected to be correlated with seeds size because the cotyledons are the only source of nutrients during the initial heterotrophic growth of the seedling. Therefore, maternal genotypes and their corresponding F_1_ progeny with similar seed weights are expected to have similar growth patterns. We also hypothesized that the genotype of the developing seedling would control traits that are more intimately associated with biochemical processes and developmental rates. To test our hypotheses, we used an information-based approach (Akaike, 1973; Burnham and Anderson, 2002) to identify the best genetic model (Tables 2, 3) that governs different aspects of early seedling growth and development of the reciprocal hybrids in relation to the maternal parents. The results from these analyses are presented below.

**Table 3:** **Estimates of root and shoot growth parameter for the best model groups**.

**Dynamic trait (Best model)**	**Parameter (unit)**	**Level**	**Estimate**	**Std. error**	**LL95%**	**UL95%**
**TOTAL ROOT LENGTH (MAPHE)**						
	*Cm* (cm d^−1^)	Large seed	149.52	6.89	135.85	163.2
		Small seed	84.86	8.6	67.8	101.93
	*Rm* (cm cm^−1^ d^−1^)	Large seed	0.92	0.11	0.71	1.13
		Small seed	0.65	0.11	0.43	0.87
	*tb* (d)	Large seed	6.22	0.18	5.86	6.57
		Small seed	6.14	0.48	5.18	7.09
**NUMBER OF BRANCHES (MAPHE)**						
	*Cm* (branches d^−1^)	Large seed	317.59	23	271.96	363.21
		Small seed	131.78	27.62	76.99	186.58
	*Rm* (branches ^−1^ d^−1^)	Large seed	1.25	0.18	0.89	1.62
		Small seed	0.74	0.17	0.41	1.07
	*tb* (d)	Large seed	6.72	0.2	6.31	7.12
		Small seed	6.37	0.79	4.8	7.95
**PRIMARY ROOT LENGTH (MAPHE)**						
	*Wf* (cm)	Large seed	26.99	1.29	24.43	29.55
		Small seed	19.64	1	17.65	21.63
	*k* (cm cm^−1^ d^−1^)	Large seed	0.26	0.02	0.23	0.3
		Small seed	0.29	0.03	0.23	0.35
	*TT* (d)	Large seed	5.51	0.24	5.04	5.98
		Small seed	3.18	0.28	2.63	3.73
**AVERAGE BASAL ROOT LENGTH (MAPHE)**						
	*Wf* (cm)	Large seed	12.93	0.81	11.32	14.54
		Small seed	6.09	0.67	4.76	7.43
	*k* (cm cm^−1^ d^−1^)	Large seed	0.33	0.02	0.29	0.38
		Small seed	0.37	0.05	0.28	0.46
	*TT* (d)	Large seed	5.84	0.17	5.5	6.19
		Small seed	4.9	0.28	4.34	5.46
**LEAF AREA (MAPHE)**						
	*Wf* (cm^2^)	Large seed	46.9	2.49	41.9	51.89
		Small seed	28.39	2.51	23.36	33.42
	*k* (cm^2^ cm^−2^ d^−1^)	Large seed	0.64	0.08	0.49	0.79
		Small seed	0.62	0.14	0.35	0.89
	*TT* (d)	Large seed	8.33	0.14	8.05	8.61
		Small seed	8.06	0.24	7.58	8.53

*LL95% and UL95% correspond to the lower and upper limit values of 95% confidence limit estimates, respectively*.

#### Dynamics of total root length (TRL)

**Figure 2:**
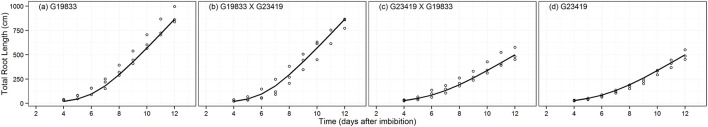
**Fitted expolinear growth function for total root length (TRL, cm) of parental genotype (A)** G19833, the reciprocal F_1_ hybrids **(B)** (G19833 × G23419) and **(C)** (G23419 × G19833), and parental genotype **(D)** G23419.

An expolinear growth function was used to model the growth patterns of TRL (Figure 2). Accordingly, an initial exponential growth phase is followed by linear growth that starts ~5–7 days after imbibition. The two parental genotypes significantly differ in total root length. The landrace G19833 had a TRL that was ~40% greater than that of the wild accession (G23419) 12 days after imbibition. The best model (Table 2) for TRL was defined by parameters associated with seed size phenotype (TRL.MAPHE, w_AICc_ = 0.71, ER = 2.5). The maximum absolute growth rate (parameter *Cm*) was approximately two fold greater (149.2 vs. 84.9 cm, respectively) for the large seeded class than for the small one (Table 3). Estimates of relative growth rates, *Rm*, tended to be greater in the large seeded genotypes than in the small seeded ones, although these differences appear to be non-significant as indicated by the partial overlap of the 95% CIs. These results indicate that seed size has an overwhelming effect on TRL as indicated by the contrasting values of the *Cm* parameter. The estimates of *Rm* are to some extent limited by the branching pattern and the number of roots (basal and tap) that have predominant growth rates. Interestingly, *tb*, the point at which the linear phase started does not seem to vary between the parental genotypes and the hybrids.

#### Dynamics of root branching pattern (FORK)

**Figure 3:**
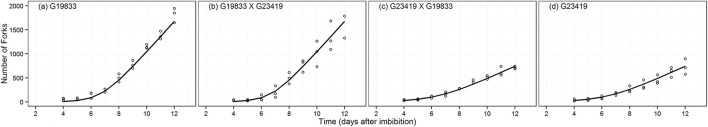
**Fitted expolinear growth function for the number of branches (fork number) data of parental genotype (A)** G19833, the reciprocal F_1_ hybrids **(B)** (G19833 × G23419) and **(C)** (G23419 × G19833), and parental genotype **(D)** G23419.

As with TRL, model testing indicated that the most parsimonious model for the branching pattern is defined by parameters estimation under seed size grouping MAPHE (Table 2: w_AICc_ = 0.94). The landrace (G19833) produced ~50% more branching points than the wild accession (G23419) (Figure 3). The absolute growth rate (*Cm*) and relative growth rate (*Rm*) were 76 and 42% higher in the large seeded group than in the small seeded one, respectively (Table 3). Interestingly, the *tb* parameter estimates showed no differences between the groups, and indicated also that the transition to the linear phase occurred almost at the same time as it did with TRL. These results indicated that branch addition followed a similar growth pattern as TRL.

#### Dynamics of primary root length (PRL) and average basal root length (AvBRL)

**Figure 4:**
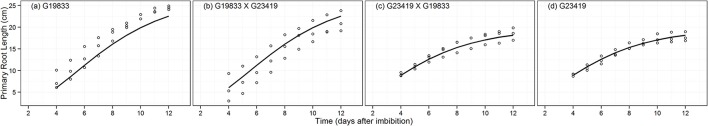
**Fitted Gompertz growth function for primary root length (PRL, cm) data of parental genotype (A)** G19833, the reciprocal F_1_ hybrids **(B)** (G19833 × G23419) and **(C)** (G23419 × G19833), and parental genotype **(D)** G23419.

**Figure 5:**
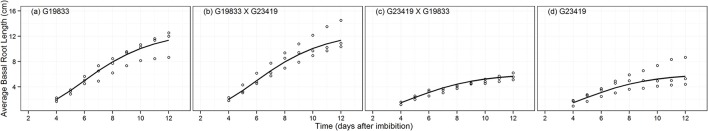
**Fitted Gompertz growth function for the average basal root length (AvBRL, cm) data of parental genotype (A)** G19833, the reciprocal F_1_ hybrids **(B)** (G19833 × G23419) and **(C)** (G23419 × G19833), and parental genotype **(D)** G23419.

The growth patterns of PRL and AvBRL were modeled using the Gompertz growth function (Figures 4, 5). As with the previous root traits, the most parsimonious model describing these patterns corresponded to the seed size grouping (MAPHE; Table 2). The large seeded group had PRLs that were 40% longer than those from the small seeded group, while the AvBRLs were over twice as long (Table 3). In both cases however, the maximum relative growth rates (*k*) of the large seeded group were 90% of those of the small group; not surprisingly, the small group attained the maximum rate earlier than the large group, 2.3 and 1.0 d for PRL and AvBRL, respectively. Another way to look at these root lengths is to express them as a function of the seed weight. Accordingly, it can be seen that while the large seeded group produces 24 cm of basal roots per gram of seed, the small seeded group produces 60 cm per gram, over twice as much. However, the investment on the primary root is much more remarkable. In this case, the large seeded group produced 50 cm of PRL for each gram of seed weight, while the small seeded group did produce 196 cm per gram. These numbers indicate that the small seeded group, as expected of most wild plants, places a premium on the primary root growth as a way to quickly reach a depth close to the water table to ensure survival.

#### Total leaf area (LAREA) dynamics, and hypocotyl (HDIA), and epicotyl diameters (EDIA)

**Figure 6:**
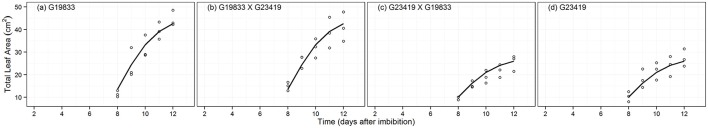
**Fitted Gompertz growth curve fitting for total leaf area (LAREA, cm^2^) of parental genotype (A)** G19833, the reciprocal F_1_ hybrids **(B)** (G19833 × G23419), **(C)** (G23419 × G19833), and parental genotype **(D)** G23419.

The growth trajectories of the leaf area were modeled using the Gompertz growth function (Figure 6). Analysis of the fitted models suggests that the most parsimonious group model was for parameters grouped according to seed size (MAPHE, Table 2). Evidence ratios in this case were > 780, suggesting that the other models tested could be discarded in favor of MAPHE. Values for the *Wf* parameter were significantly different for large and small seeded genotypes (Table 3). It further suggests that the large-seeded reciprocal F_1_ hybrid and its corresponding maternal parent were capable of producing ~60% larger leaves than the small-seeded genotypes. However, the estimated value for relative rate parameter *k* and time for maximum relative rate (*TT*) were much similar for both groupings (Table 3).

Epicotyl and hypocotyl diameters of the parental seedlings differed by a factor of two. In addition, each of the reciprocal hybrid class had diameters that were indistinguishable from those of the maternal parents. These results again are similar to those previously found for leaf and root size traits, where seed size appears strongly positively associated with final organ size.

### Comparative resource allocation model based on seed reserves remobilization and early seedling development

The results presented above clearly indicated that stored energy reserves in cotyledons have a significant effect on the size of early seedling organs. This effect appeared to be exerted through a combination of the reserve remobilization rate and the assimilation rate of the remobilized reserves. The kinetics of these activities was evaluated in all genotypes by monitoring changes in dry weights over time using destructive sampling.

#### Decay of cotyledon energy reserves

**Figure 7:**
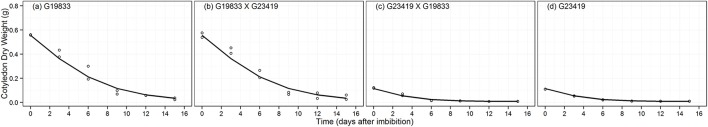
**Fitted decay curve for cotyledon dry weight decay (CDW, g) over time of parental genotype (A)** G19833, the reciprocal F_1_ hybrids **(B)** (G19833 × G23419) and **(C)** (G23419 × G19833), and parental genotype **(D)** G23419.

The trajectories of cotyledon dry weight (CDW) decay conformed to the MAPHE model representing the two seed size classes (Table 2 and Figure 7). This model presented very high evidence ratios (>1190), indicating that the remaining models contribute little to understanding the decay of cotyledon reserves. There was an approximately fourfold difference in initial seed mass between the two seed classes as shown by the *Max* parameter, while the *Min* parameter also significantly differed between the two seed size classes (Table 4). The decay function captured values that reflected the relative sizes of the planted seeds as shown in Table 1. It must be pointed out that dry weight data were obtained without the seed coat, which represents approximately from 5 to 10% of the seed dry weight. Once these values are corrected by including the seed coat, the 95% confidence limits of the estimated MAX values encompass the observed values. As expected, the time to reach the mid-point (*TT*) between the *Max* and *Min* cotyledon dry weights was significantly different between the two maternal seed size groups (Table 4).

**Table 4:** **Estimates of best model parameters for decay in cotyledon dry weight (CDW, g), and seedling dry weight accumulation (SDLDW, g)**.

**Best model**	**Parameter (unit)**	**Level**	**Estimate**	**Std. error**	**LL95%**	**UL95%**
**DECAY IN COTYLEDON DRY WEIGHT (MAPHE)**						
	*Max* (g)	Large seed	0.436	0.015	0.406	0.465
		Small seed	0.121	0.057	0.009	0.232
	*Min* (g)	Large seed	0.058	0.004	0.050	0.066
		Small seed	0.011	0.004	0.004	0.019
	*TT* (d)	Large seed	6.012	0.192	5.636	6.387
		Small seed	2.727	0.951	0.863	4.590
**SEEDLING DRY WEIGHT ACCUMULATION (MAPHE)**						
	*Wf* (g)	Large seed	0.55	0.04	0.47	0.63
		Small seed	0.19	0.04	0.1	0.28
	*k* (g g^−1^ d^−1^)	Large seed	0.26	0.02	0.22	0.31
		Small seed	0.24	0.06	0.12	0.36
	*TT* (d)	Large seed	7.28	0.33	6.62	7.95
		Small seed	7.35	1.16	5.01	9.69

*LL95% and UL95% correspond to the lower and upper limit values of 95% confidence limit estimates, respectively*.

#### Seedling dry weight accumulation

**Figure 8:**
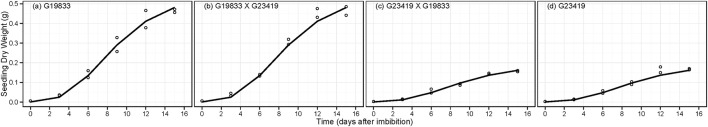
**Fitted Gompertz growth equation for seedling dry weight accumulation (SDLDW, g) over time for parental genotype (A)** G19833, the reciprocal F_1_ hybrids **(B)** (G19833 × G23419) and **(C)** (G23419 × G19833), and parental genotype **(D)** G23419.

The analysis yielded an evidence ratio (ER > 2437; Table 2) that dictated selection of the MAPHE model to the exclusion of the others. The accumulation of seedling dry weight (SDLDW) followed an initial lag phase that lasted approximately the three first days, a maximum growth phase observed between 3 and 12 days, and a final asymptotic phase that started 12 days after imbibition (Figure 8). Interestingly, of the three Gompertz parameters estimated for the two groups, only the final seedling dry weight (*Wf*) showed significant differences, the large seed size group having approximately threefold greater DW than the small seeded group (0.55 vs. 0.19 g) (Table 4). In contrast, neither the maximum relative growth rate (*k*, g g^−1^ d^−1^) nor the time to reach maximum relative growth rate (*TT*, days) differed significantly between the seed size classes.

## Discussion

The objective of this project was to study root growth and development of young seedlings in the common bean, and to distinguish the effects of the seed size phenotype from those controlled by embryo genotype. The fact that in this species the maternal parent controls the size of the seed it produces, regardless of the pollen donor, offered a potential model to distinguish the two effects using reciprocal F_1_ hybrids of parents with contrasting seed weights. This was considered a valid approach, provided that the genotype of the seedlings is fully expressed during early seedling growth. However, the results presented here suggest that this may not be the case.

Seed growth and development is complex due to the heterogeneous genotypic makeup of seed tissues. An example of this complexity is the maternal control of seed weight and morphology observed in reciprocal F_1_ hybrids of the common bean (Figure 1 and Table 1). Studies in legumes have shown that following fertilization, cell division of the seed coat (maternal), and the endosperm (filial) precedes cell division in the embryo (filial), which ensues after division has slowed down in the previous tissues (Hedley and Ambrose, 1980; Goldberg et al., 1989, 1994). At the same time, the seed coat (maternal) produces the metabolic signals that control growth of the endosperm and the embryo, both of which are filial tissues (Weber et al., 1995, 1996). Furthermore, Weber et al. (1996), Coello and Martinez-Barajas (2014), and Lemontey et al. (2000) argue that the developmental program executed in the seed coat controls the duration of the cell division phase and consequently the final size of the seed as this is correlated with cell number as shown by Davies (1975) in *Pisum*. In contrast, the second phase of seed development encompasses the absorption of the endosperm, cell expansion in the embryo tissues and accumulation of reserves. These phenomena could explain the maternal control of seed size observed in the reciprocal F_1_ hybrids.

Also, the results presented here resemble those reported by Jofuku et al (2005) with Arabidopsis crosses involving APETALA2 mutants, as far as the seed weight phenotype is concerned. Their work showed that the maternal allele of this transcription factor has an effect on seed weight by acting in sporophytic and endosperm tissues. The latter may be explained by imprinting, the differential expression of maternal or paternal alleles (Rodrigues and Zilberman, 2015). In fact, the endosperm of different species displays extensive imprinting (Rodrigues and Zilberman, 2015, and references therein), and this phenomenon can explain the action of the Apetala2 maternal allele as reported by Jofuku et al (2005), and the results presented here as well. Developmentally controlled gene expression in the maternal seed coat in combinations with epigenetic phenomena in the endosperm may explain the maternal control of seed size. However, maternal control of the seed shape phenotype (Table 1) is difficult to explain by metabolic control alone, but could be explained by maternal imprinting in the embryo, although imprinting in the embryo has been reported to be uncommon and sporadic (Raissig et al., 2011; Pignatta et al., 2014). In summary, developmentally controlled gene expression of the maternal seed coat in combinations with epigenetic phenomena may explain the maternal control of seed size and morphology in the reciprocal F_1_ hybrids.

Growth analysis of the reciprocal F_1_ hybrid seedlings indicated that the maternal effects on seed size and morphology appeared to extend to seedling growth. However, this phenomenon has been documented long ago in a few species (See review by Roach and Wulff, 1987), but references are only made about the size or dry matter accumulation of the seedlings, which could be influenced by the size of the seed. To sort out seed size from embryo-genotype effects on different seedling growth characteristics, the AIC-based approach to parameter estimation was used to identify the model that best represented the observed data. Analysis of the results presented here showed that parameter estimates for organ size (roots, stems, and primary leaves) of the non-linear models (e.g., *Wf* of the Gompertz function) clearly group the reciprocal F_1_ seedling size-phenotypes with those of the corresponding maternal parent. These results are not surprising because the cotyledons, where reserves are stored, represent at least 90% of the seed weight, and large seeds can fuel growth of the seedling to a greater extent than small seeds can. This effect was further confirmed with the analysis of resource remobilization where large seeds produced larger seedling. However, final seedling dry weight on the basis of initial seed weight favored the small seeded group (1.27 vs. 1.57) suggesting the latter has a more efficient mechanism.

Another aspect of the findings presented here is that although seed size/weight may have been the primary target of domestication of the common bean, selecting for larger seeds had a major agricultural significance because larger seeds can produce larger seedlings with a greater competitive ability so as to ensure a good stand establishment.

Interestingly, the results presented here also revealed strong similarities in morphology parameters between the reciprocal F_1_ seedlings and their respective maternal parents. For example, root branching patterns (number and rates) were significantly different between seed size classes. Root branching is a key component of root architecture, a character more closely controlled by gene action (Svistoonoff et al., 2007; Thomann et al., 2009), than by resource supply. In addition, the preferential allocation of reserves to the taproot in the small seeded group suggests that the genetic control of developmental and morphological characteristics was very similar within each seed size group.

Almost all the crosses we have made in the lab throughout the years have shown the maternal effect on seed size observed in legumes and many other species. However, the maternal effect on morphological traits detected in reciprocal hybrids from a single parental pair can't be assumed to be a general phenomenon until a more comprehensive analysis is carried out. For instance, Davies (1975) carried out a full diallel mating experiment with multiple garden pea accessions. In this experiment he showed that not all crosses displayed maternal effect on seed weight. Also, F_1_ hybrids and backcross progenies between a cultivated bean and a breeding line carrying multiple chromosome segments introgressed from *P. coccineus* did not display maternal effects on seed size (Vallejos and Chase, 1991). Genetic variation in this characteristic offers the opportunity for future investigation of the mechanism that governs this phenomenon, including whether the maternal effect on seed size and on seedling morphological characteristics are connected.

In summary, a comparative analysis of reciprocal F_1_ hybrids of the common bean showed strong and significant maternal effects on both seeds and seedlings. Seed weight and seed morphology measurements could not distinguish between seeds produced by selfing from those produced by hybridization with pollen from a paternal parent with drastically different seed weight and shape. This phenomenon has been reported for many species including pea (Davies, 1975) and Arabidopsis (Jofuku et al, 2005), among others. However, comparisons of growth and developmental patterns of the reciprocal F_1_ hybrids showed two types of maternal effects. One of these has been reported in the literature (Lemontey et al., 2000) and refers principally to the maternal effect on seedling growth. This phenomenon appears to be a direct consequence of the maternal effect on seed size—larger seeds produced larger seedlings. Thus, a seedling size is largely proportional to the reserves stored in the seeds and it is not necessarily a reflection of the genotype of the seeds. The second maternal effect on the seedlings was detected by root morphology measurements; for instance, the relative tap-root length or the branching patterns. This type of effect is difficult to ascribe to the amount of seed reserves, and more likely to be explained by parent-of-origin specific gene expression in the embryo. Whether this phenomenon is due to parental imprinting or established patterns for the timing of zygote genome activation remains to be determined. Both are burgeoning areas of research that have primarily focused on few model organisms (Baroux et al., 2008; Autran et al., 2011; Nodine and Bartel, 2012; García-Aguilar and Gillmor, 2015). Although Davies (1975) reported maternal control of seed size as common in the garden pea, he also detected notable exceptions in a full diallel experiment. Furthermore, genetic variation for maternal control of seed size has also been detected in *P. vulgaris* (Vallejos and Chase, 1991). This type of variation raises the possibility of a genetic characterization of this mechanism without the need of induced mutations.

## Author contributions

JS, SG, and CV conceived and designed the experiments. JS and HL conducted the experimental work. JS, SG, and JM carried out the statistical analyses. JS, SG, JM, and CV analyzed and interpreted the results. JS and CV prepared the manuscript with input from SG, JM, and all the authors read and approved the final version of the manuscript.

## Funding

This work was supported in part by a grant from the National Science Foundation IOS-0920145.

### Conflict of interest statement

The authors declare that the research was conducted in the absence of any commercial or financial relationships that could be construed as a potential conflict of interest.
