# Supplementary material for: Maternal Effects on Seed and Seedling Phenotypes in Reciprocal F1 Hybrids of the Common Bean (Phaseolus vulgaris L.)
Source: Front Plant Sci. 2017 Jan 24;8:42. doi: 10.3389/fpls.2017.00042 (PMC5259735; doi:10.3389/fpls.2017.00042)
Supplement: Supplementary file 1 [file DataSheet1.DOCX]

**Maternal Effects on Seed and Seedling Phenotypes in Reciprocal F_1_ Hybrids of the Common Bean (*Phaseolus vulgaris* L.)**

Authors: **Jugpreet Singh, Jose A. Clavijo Michelangeli, Salvador A. Gezan, Hyungwon Lee, C. Eduardo Vallejos**


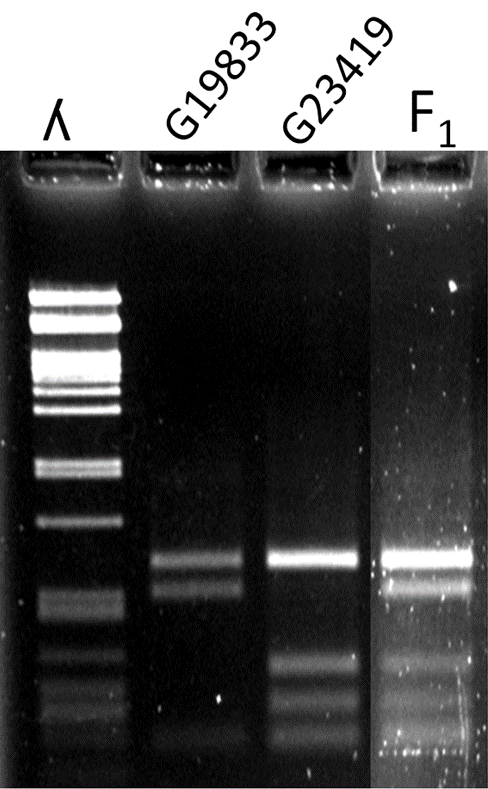


**Figure S1.** DNA marker analysis of F_1_ hybrids. PCR amplification products of AP2 from the parental genotypes and the F_1_ hybrids were digested with *Taq*I and the fragments separated in an agarose gel.
